# Effects of Saffron (*Crocus sativus *L.) Stigma Extract and its Active Constituent Crocin on Neuropathic Pain Responses in a Rat Model of Chronic Constriction Injury

**Published:** 2016

**Authors:** Hossein Ali Safakhah, Tahereh Taghavi, Ali Rashidy-Pour, Abbas Ali Vafaei, Mina Sokhanvar, Narges Mohebbi, Mostafa Rezaei-Tavirani

**Affiliations:** a *Laboratory of Pain Research, Research Center and Department of Physiology, Faculty of Medicine, Semnan University of Medical Sciences, Semnan, Iran.*; b *Proteomics Research Center, Shahid Beheshti University of Medical Sciences, Tehran, Iran*

**Keywords:** Saffron (*Crocus sativus *L.), Crocin, Neuropathic pain, Allodynia, Hyperalgesia, Rat

## Abstract

This study was designed to investigate the therapeutic effects of saffron (*Crocus sativus *L.**)** and its main constituent crocin on neuropathic pain behavioral responses induced by chronic constriction injury (CCI) in rats. Adult male Wistar rats (200 to 250 g) were randomly assigned into 5 groups: Sham + saline, CCI + saline, CCI+ saffron (30 mg/kg), CCI + crocin (15 mg/kg) and CCI + crocin (30 mg/kg). CCI was induced by applying 4 loose ligatures around the sciatic nerve. Two weeks after nerve lesion, injections of saline, saffron or crocin were started and continued until 26^th^ day post-surgery. Pain behavioral responses including mechanical allodynia (von Frey filament testing) and thermal hyperalgesia were measured in 14, 17, 20, 23, 26, and 40^th^ days after CCI. CCI significantly increased pain behavioral responses. Saffron and crocin (30 mg/kg) decreased thermal hyperalgesia and mechanical allodynia on day 26, and this effect continued until the day 40. Crocin at lower dose (15 mg/kg) was ineffective. These findings indicate that treatment of saffron and crocin after CCI may have a therapeutic effect against neuropathic pain, suggesting that these substances may offer new strategies for the treatment of this highly debilitating condition.

## Introduction

Neuropathic pain is caused by damage or disease that affects the structure and functioning of the somatosensory system. As a result, aberrant pains and pathological activities may occur spontaneously or as amplified responses to noxious and innocuous stimuli (1). This persistent pain induces further peripheral or central nervous system complications (2). Peripheral neuropathic pain found in physical trauma to a nerve trunk, abnormal neuronal metabolism, infections, harmful effects of neurotoxic agents, and tumor invasions causes multiple pathophysiological alterations in both the peripheral or central nervous system (3). Neuropathic pain is mostly accompanied by two classic behavioral parameters: hyperalgesia (an increased sensitivity to painful stimuli) and allodynia (a pain due to a stimulus which does not normally provoke pain). These two parameters are abnormal sensory signs which can be seen in cases with neuropathic pain (2). 

The mechanisms of neuropathic pain are complex and less known (4). Today, the most prescribed medicines have unintended and undesirable adverse effects and most patients are often unsatisfied. Consequently, findings more suitable medicines are essential for effective treatment and care of neuropathic patients (5). Presently, antidepressants and anticonvulsants are the first choice for the treatment of patients with neuropathic pain; however, these medicines produce only partial relief and they accompanied by many adverse effects (6). Consequently, the management of neuropathic pain needs special attention and today finding new medicines is an interesting topic for researches.

Medicinal plants have been used in treating many disorders from centuries ago (7). As the ingredients existing in herbal medicines have less adverse effects, more energy can be put into testing medicines with herbal origins for treatment of neuropathic pain in the pharmaceutical sciences. *Crocus Sativus* L, known as saffron, is an aboriginal plant growing in various parts of the world, particularly in Iran. Crocin, picrocrocin, and safranal are three major biologically active ingredients of saffron (8). Saffron also is a rich resource of mineral and trace elements (9). In traditional herbal medicine, saffron is used for depression, fear, sleep disorders, pain relief, asthma, cardiovascular diseases, etc (10). During the last decade, findings from several laboratories have shown that saffron and its components including crocin and safranal have anti-tumor (11), neuroprotective (12), analgesic and anti-inflammatory (13) anti-stress and enhancer spatial learning and memory effects (14). Crocin and safranal have chemo-preventive and geno-protective effects as well. They also protect from genotoxin-induced oxidative stress and methyl methanesulfoate-induced DNA damages in labs mice (15). Additionally, it is found that the apical and the lateral bud of *Crocus sativus* L. have notable anti-nociceptive and anti-inflammatory activities (16). Neuro-protective effects of saffron are also proven by other similar studies (12).

Hosseinzadeh and Younesi (2002) tested the anti-inflammatory effects of aqueous and ethanolic extracts from saffron and Crocus flowers in mice, using the hot plate and writhing tests. Whereas no effect was found in the hot plate test, the stigmata inhibited the acetic acid induced writhing reflex. The effect could only partially be inhibited by naloxone (13). Also previous studies indicated that ethanolic and aqueous extract of saffron and crocin had anti-allodynia and anti-inflammatory effects in different animal models (13, 17, 18). As the main volatile constitution of saffron, safranal has also been demonstrated antiallodynia effect in the formalin, acetic acid or carrageenan induced acute pain, and sciatic chronic constriction induced neuropathic pain (18, 19). In rodents, it was shown that safranal has low-toxic effects when adiministrated intraperitoneally (20). 

Chronic constriction injury (CCI) of the sciatic nerve in rats is an animal model of neuropathic pain that produces disorders of pain sensation like those seen in man. These rats represent the patients with signs or symptoms of neuropathic pain including allodynia, mechanical and thermal hyperalgesia, extraterritorial pain, and guarding behavior happening in spontaneous pain (21). Since saffron has anti-nociceptive and anti-inflammatory activities (13), it may be useful in reducing the resulting pain behaviors in the rat CCI model. As common clinical observations indicated that neuroprotective interventions should be performed early after injury, it may also be suggested that beginning of saffron or crocin treatment before establishing pain hypersensitivity would be effective. Therefore, the present study was undertaken to examine the effect of intermediate initiation of chronic and systemic administration of saffron and its main constituent crocin on expression and development of hyperalgesia and allodynia behaviors in the rat CCI model of neuropathic pain.

## Experimental

*Animals*

Adult male Wistar rats (200-250 g) were individually housed in cages (50 × 26 × 25 cm) and kept on a 12-h light/dark cycle (6 am lights on–6 pm lights off) with food and water available ad libitum. The ambient environment was maintained at constant temperature (22 ± 2 ºC) and relative humidity (50 – 60 %). All animal treatments were conducted in accordance with the National Institutes of Health Guide for the Use and Care of Laboratory Animals and were approved by the local ethical committee. In addition, care was taken to minimize the number of animals that were used in each experiment. Three days before any behavioral testing, the rats were kept in their testing room for about two hours each day. During this time, rats were alternatively handled by the investigator.

Pure red saffron powder was kindly supplied by the Pharmaceutical Research Center, Mashhad University of Medical Sciences, Mashhad, Iran (The specimen number of the plant is 134-0319-1). Crocin was purchased from Sigma Aldrich. Both substances were dissolved in a physiological saline and injected intrapritonealy in a volume of 1 mL/kg. Saffron extract at a dose of 30 mg/kg and crocin at doses of 15 and 30 mg/kg were injected for 14 days in treatment groups. These doses were chosen on the basis of our pilot studies and previous reports (14, 22, 23). 

*CCI induction*

CCI was produced by loose ligation of the sciatic nerve as previously described (21). Animals were anesthetized using 50 mg/kg ketamine-rompan (1:8 ratio) and included left sciatic nerve exposition at mid-thigh level by blunt dissection through the biceps femoris muscle. Four constrictive ligatures (4–0 chromic gut suture) were loosely tied around the nerve at distances of about 1 mm. Muscle and skin were closed in layers. In the sham group, operations were performed to expose and mobilize the nerve but there was no ligation. All operations were done by the same person to minimize differences in method. 

*Experimental groups*

**Figure 1:**
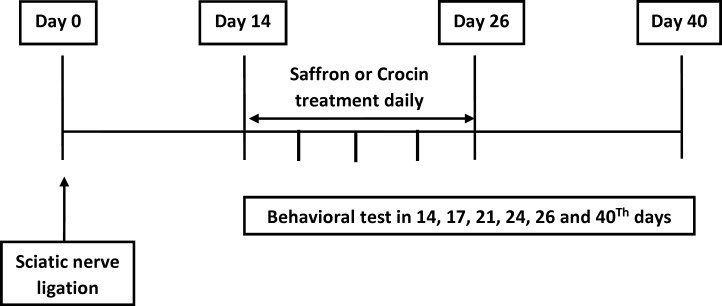
Time line of experimental procedures (see Materials and methods for details

Rats were randomly divided into five experimental groups (each, n = 11) as follows: Sham + Saline, CCI + Saline, CCI + Saffron (30 mg/kg), CCI + Crocin (15 mg/kg), and CCI + Crocin (30 mg/kg), and in all groups, drug or saline was given 30 min before behavioral tests daily for 14 days post-surgery. Next, behavioral tests including the thermal hyperalgesia, and mechanical allodynia were performed in each group on days of 14, 17, 20, 23, 26 and 40 after CCI (Figure 1). 

*Behavioural tests*

For testing mechanical allodynia (von-Frey filament testing), the animals were placed in a Plexiglas box (20 × 20 × 30 cm) with a mesh floor based on the method described by Pitcher *et al.* in their 1999 study (24). Each rat was placed in the testing chamber and permitted to acclimatize for 30 min prior to testing. Von Frey filaments (Stoelting Co, Wood Dale, IL) in scores of 2, 4, 6, 8, 10, 15, 26, and 60 g were applied to the plantar soft tissue of the hind paw to determine the withdrawal threshold. The first filament applied matched to a force of 2 g. Each filament was applied three times, each for three seconds, and at intervals of five seconds. If a negative response (no movement) was seen, the filament exerting the next greater force was used. If two of the observed responses were positive (paw withdrawal), the obtained score was considered a response threshold. If the animal did not respond to the score of 60 g, that score was considered a threshold response. 

For thermal hyperalgesia, paw-withdrawal latency to a thermal nociceptive stimulus was assessed as described elsewhere (25). Rats were placed in a Plexiglas box (20 × 15× 15 cm) on top of a glass floor (Plantar test No. 7370; UgoBasile, VA, Italy) and allowed to acclimate for 30 min before testing. Then, a mobile infrared radiant heat source (infrared intensity 60) was placed under the glass floor and focused onto the right hind paw (intact) or the left (ligated) hind paw. A digital timer recorded the paw-withdrawal latency. Throughout the whole experiment, stimulus intensity (infrared 60) was kept constant. Each rat was tested in three sequential trials with an interval of 10 min, and the recorded paw-withdrawal latencies were averaged. Difference scores were calculated by subtracting the mean withdrawal latency of the right (intact) side from the left (ligated) side. To avoid possible tissue injury, a cutoff time of 60 s was applied.

*Statistical analysis*

Data are presented as mean ± SEM. Mechanical allodynia and thermal hyperalgesia data was analyzed by a two way repeated measure analysis of variance (ANOVA) followed by Tukey’s post-hoc test. Statistical differences were considered significant when P<0.05.

## Results

The majority of the nerve ligated rats appeared healthy and well-groomed. Paw gesture of the ipsi-lateral paw was slightly altered, but this did not interfere with the normal daily activities of the rat. We observed behavioral symptoms which developed simultaneously, including hyperalgesia, spontaneous pain, abnormal gait, and weight bearing on the opposite side of the operated limb. 

**Figure 2:**
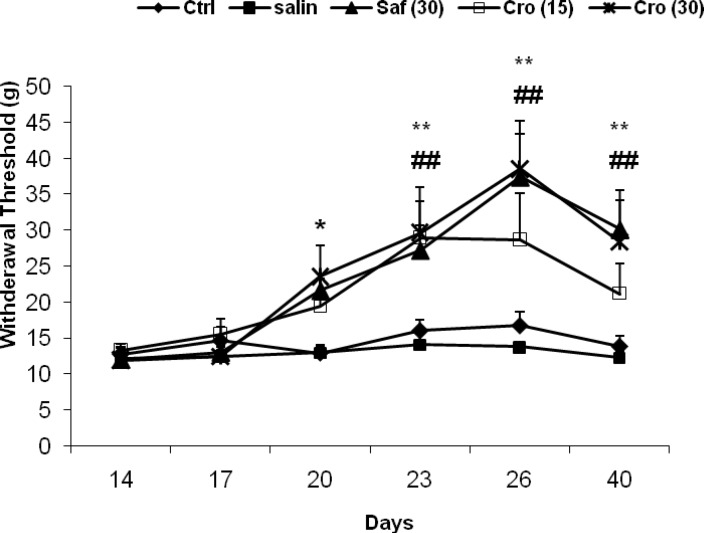
Effects of chronic uses of saffron extract and crocin on mechanical allodynia – induced by CCI. Data are expressed as the mean ± SEM. ^b1^P = 0.007; ^b2^P = 0.005, ^c1^P = 0.001, and ^c2^P = 0.004 as compared with the CCI + Saline group at the same day. C: crocin; SE: saffron extract

The results of the behavioral tests for the mechanical allodynia are shown in Figure 2. After ligation of the sciatic nerve, the injured hind paw became sensitive to mechanical stimuli, even with the weaker von-Frey filament testing. There was a significantly enhanced response to the stimulus in the CCI rats compared with the sham animals as measured on any testing day's post-CCI and saffron or crocin treatment prevented this effect on days 26 and 40. A two way ANOVA (tests ×groups) with repeated measurement revealed significant effects of groups (F_5, 50 _= 11.59, P = 0.0001), significant effects of days (F_5, 250_ = 13.14, P = 0.0001) and a significant interaction between both factors (F_20, 250_ = 2.36, P = 0.001). Post-hoc comparisons showed a significant difference between the Sham + Saline group with the CCI + Saline group at all testing days (Ps ranging <0.05 to <0.0001). On days 26 and 40, there were significant differences between the CCI + Crocin (30 mg/kg) the CCI + Saline group (day 26: P = 0.029; day 40: P = 0.035), and between the CCI + Saffron and the CCI + Saline (day 26: P = 0.014; day 40: P = 0.028).

**Figure 3:**
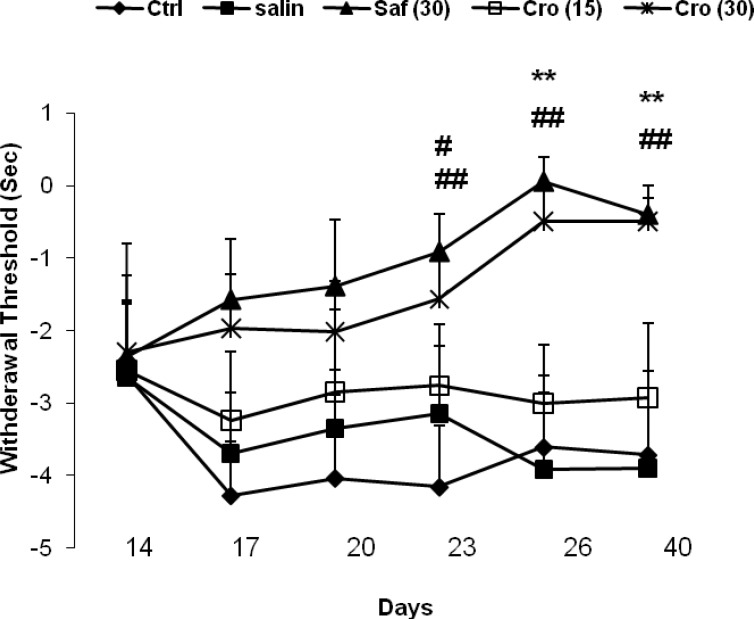
Effects of chronic systemic administration of saffron extract and crocin on thermal hyperalgesia-induced by CCI in rats. Data are expressed as the mean ± SEM. ^b1^P = 0.029; ^b2^P = 0.035, ^c1^P = 0.014, and ^c2^P = 0.03 as compared with the CCI + Saline group at the same day. C: crocin; SE: saffron extract

The results of the behavioral tests for the thermal hyperalgesia are shown in Figure 3. The loose ligation of the sciatic nerve caused a significant decrease in the paw withdrawal latency to the thermal stimulus in the injured limb of CCI rats in comparison with sham rats as measured on any of testing day's post-CCI, and saffron or crocin treatment prevented this effect on days 26 or 40. A two- way ANOVA (days × groups) with repeated measures on days revealed significant effects of groups (F_4, 50 _= 5.67, P = 0.001), no significant effects of days (F_5, 250 _= 0.95, P = 0.45) and no significant interaction between both factors (F_20, 250_ = 0.89, P = 0.61). Post-hoc comparisons showed a significant difference between the Sham + Sal group with the CCI + Saline at all testing days (Ps ranging <0.05 to <0.001) except on day 14. On days 26 and 40, there were significant differences between the CCI + Crocin (30 mg/kg) and the CCI + Saline group (day 26: P = 0.007; day 40: P = 0.005), and between the CCI + Saffron and the CCI + Saline (day 26: P = 0.001; day 40: P = 0.004).

## Discussion

The aim of this study was to examine the effects of chronic systemic injections of saffron and crocin on neuropathic pain behaviors in the rat CCI model. We found that saffron and its main constitute crocin are able to alleviate both mechanical allodynia and thermal hyperalgesia. This effect was long-lasting and continued up 40^th^ day post-surgery, suggesting that these substances could have a therapeutic effect in the management of neuropathic pain. 

CCI model is one of the best defined behavioral models for neuropathic pain in rats. This model is the most used experimental model of peripheral nerve injury equivalent to clinical neuropathic pain (21). Our data clearly demonstrated that repeated saffron or crocin treatment started before full establishment of neuropathy (14 days after the model induction) and continued for 13 days, effectively mediate reversal of pain behaviors in one of the well characterized models of neuropathic pain, the CCI model. In our experimental design, following establishment of a strong neuropathic hypersensitivity in day 14 (21), daily systemic injections of saffron or crocin was started and continued over 16 days. Behavioral measurements were done in days 14, 17, 20, 23, and 26. In order to exclude immediate analgesic effects and to focus on long lasting effects of these substances, we also measured the behavioral responses in day 40 after the surgery (two weeks after the last injection). Thus, the effects of intermediately repeated administration of saffron or crocin do not appear to be due to an analgesic effect. For the reason that saffron and crocin promotes recovery of tactile and thermal hypersensitivity with long-term treatment during the intermediately phase of model development, it may be postulated that saffron amd crocin may be having a neuroprotective and/or therapeutic effect in this phase of CCI model.

Our findings support the results of recent studies suggesting that aqueous extracts of saffron and safranal diminish thermal and mechanical hyperalgesia (17), and ethanolic and aqueous extracts of *Crocus sativus L.* stigma attenuate oxidative stress, inflammation and apoptosis in CCI rat model (26). In the former study, it was shown that systemic injection of saffron and its constituent safranal, but not crocin one day before surgery and up to 10 days after surgery significantly alleviated the behavioral manifestations of neuropathic pain. These findings suggest the preventive effects of saffron and safranal on neuropathic pain. Since during these times, neuropathic pain responses are not still fully established (27), the observed beneficial effects of saffron and crocin may indicate a preventive rather therapeutic effect. In our study, we injected saffron and crocin at day's 14-26 post-CCI, when neuropathic pain is already established. Our findings indicated that the effects of saffron and crocin on mechanical allodynia and thermal hyperalgesia were seen from 26^th^ day post-CCI that continued to 40^th^ day. Thus, the beneficial effects of these substances may indicate their therapeutic effects. The main difference of the present study and these two studies is the duration of drug administration (40 days vs 10 days), i.e chronic administration of the extract and crocin. The other difference is the doses of the extract and crocin which were much lower in the present study than previous studies. In addition, the previous study failed to show any effect for crocine even with higher concentration which is due to chronic administration. In fact, the effect of crocine was appeared 17 days after its administration. 

The exact mechanism(s) of anti-neuropathic pain effects of saffron has not been clarified yet; however, it seems different pathways contribute in order to leave the beneficial effects of saffron. Our knowledge of phytochemical effects of plants, based on what formerly are found, suggested that the antinociceptive and anti-inflammatory effects of the saffron extract may be due to their flavonoids, tannins and anthocyanins. Similar studies have demonstrated that flavonoids such as rutin, quercetin, luteolin, hesperidin and biflavonoids induce remarkable anti-nociceptive and/or anti-inflammatory activities (28, 29). A few recent reports have discussed the antinociceptive and anti-inflammatory activities of tannins and crocins in some models of inflammation (30) (31). Hence, it can be realized that effects of saffron extracts on neuropathic pain could be due to their flavonoids, tannins, anthocyanins, alkaloids, and saponins.

One possible mechanism that may underline the inhibitory effects of crocin against neuropathic pain is reduction of intracellular calcium. Crocin inhibits the extracellular Ca^2+ ^influx and releases intracellular Ca^2+^ stores in the endoplasmic reticulum (32). Reduction of intracellular Ca^2+^ release can cause relaxation of blood vessels leading to hyperemia in tissue (33) which possibly is beneficial to reduce neuropathic pain. In addition, safranal can cause changes in GABA_A_-benzodiazepine receptor complex (34). Benzodiazepines, in pre-anesthetic doses, reduce blood pressure by decreasing peripheral resistance or cardiac output (35). Safranal affects on GABA_A_-benzodiazepine receptor complex may contribute to its effect on blood pressure in control of neuropathic pain.

oxidative stress as well as production of free radicals has been considered to be involved in the pathogenesis of neuropathic pain (36). There is convincing evidence confirming that antioxidant agents have an essential role in reducing neuropathic pain. An in vitro study has shown that ethanolic and aqueous extracts of saffron as well as crocin and safranal (to a lesser extent) induces significant anti-oxidant activities by decreasing lipid peroxidation rate by means of Fe^2+^ absorbed via reduction of malondialdehyde (MDA). They also can prevent from lipid peroxidation by inhibiting deoxyribose degradation in erythrocyte membrane and liver microsomoes. Recently, we have demonstrated that both saffron and crocin can prevent the oxidative damage to the hippocampus and cognitive impairments by chronic stress (14). Thus, it can be assumed that the inhibitory effects of saffron and crocin on pain behaviors in the rat CCI model, at least in part, are associated with their antioxidant activity.

In many neurodegenerative diseases like neuropathic pain, the accumulation of excessive glutamate is found in the extracellular space (37). An abnormally high level of glutamate can lead to neurotoxicocity. Blocking the glutamate actions by specific antagonists have been shown to induce neuroprotective effects (38). A recent study has shown that acute systemic injection of safranal, a main component of saffron, reduces the extracellular concentrations of glutamate and aspartate in the rat hippocampus following kianic acid (KA) administration, indicating a protective effect of this substance against KA-induced neural damage (39). This protective effect may play a role in the inhibitory action of saffron on neuropathic pain as found in the current study.

There were some limitations in this study like using the narrow range of saffron extract and crocin does. Another limitation is the lack of measurement of the amount of crocin present in the saffron samples. These aspects should be considered in future studies on the effects of crocin or saffron on neuropathic pain using different models and conditions.

Findings of this study showed that chronic administration of saffron and crocin reduces the neuropathic pain responses in the rat CCI model, suggesting these substances could have potential therapeutic applications in the treatment and management of neuropathic pain in humans. Further studies are required to determine the underlying mechanisms of inhibitory influences of these substances on neuropathic pain.

*Authors’ contributions*

TT, MS, and NM carried out animal experiments, HAS, participated in design of the study, contributed to the experiments, performed the statistical analysis, and helped draft manuscript. AAF wrote the early version of the manuscript. ARP conducted the study and edited the final version of manuscript. All authors read and approved the final manuscript.
